# Maltreatment, the Oxytocin Receptor Gene, and Conduct Problems Among Male and Female Teenagers

**DOI:** 10.3389/fnhum.2018.00112

**Published:** 2018-03-22

**Authors:** Dimitrios Andreou, Erika Comasco, Cecilia Åslund, Kent W. Nilsson, Sheilagh Hodgins

**Affiliations:** ^1^Department of Clinical Neuroscience, Karolinska Institute, Stockholm, Sweden; ^2^1st Department of Psychiatry, National and Kapodistrian University of Athens, Athens, Greece; ^3^Science for Life Laboratory, Department of Neuroscience, Uppsala University, Uppsala, Sweden; ^4^Centre for Clinical Research, Department of Neuroscience, Uppsala University, Västerås, Sweden; ^5^Institut Universitaire en Santé Mentale de Montréal, Université de Montréal, Montreal, Canada

**Keywords:** oxytocin receptor gene, rs53576, maltreatment, conduct problems, gene–environment interaction

## Abstract

The oxytocin receptor gene (*OXTR*) influences human behavior. The G allele of *OXTR* rs53576 has been associated with both prosocial and maladaptive behaviors but few studies have taken account of environmental factors. The present study determined whether the association of childhood maltreatment with conduct problems was modified by *OXTR* rs53576 genotypes. In a general population sample of 1591 teenagers, conduct problems as well as maltreatment were measured by self-report. DNA was extracted from saliva samples. In males, there was a significant positive association between maltreatment and conduct problems independent of the genotype. In females, among G allele carriers, the level of conduct problems was significantly higher among those who had been maltreated as compared to those not maltreated. By contrast, among female AA carriers, conduct problems did not vary between those who were, and who were not, maltreated. The results indicate that *OXTR* rs53576 plays a role in antisocial behavior in females such that the G allele confers vulnerability for antisocial behavior if they experience maltreatment, whereas the A allele has a protective effect.

## Introduction

Oxytocin is a highly evolutionarily conserved nonapeptide, synthesized in the paraventricular and the supraortic nucleus of the hypothalamus. Oxytocin has been associated with prosocial behavior in both animal and human studies and has therefore been characterized by the popular press as ‘the love hormone.’ For instance, increased endogenous plasma oxytocin concentrations have been associated with increased trust (Kosfeld et al., 2005) and empathy (Barraza and Zak, 2009), while intranasal administration of synthetic oxytocin has been associated with improved mentalizing (Domes et al., 2007) and face emotion recognition (Van IJzendoorn and Bakermans-Kranenburg, 2012).

Oxytocinergic neurotransmission is mediated by the oxytocin receptors (OXTRs) that are highly expressed in the limbic system, mainly in the ventral striatum and amygdala (Vargas-Martinez et al., 2014). The oxytocin receptor gene (*OXTR*) is located on chromosome 3p25 and *OXTR* gene variation may modulate the signaling properties of the OXTRs. The best studied *OXTR* gene variant is a G/A single nucleotide polymorphism (SNP), rs53576, located in the third intron of the gene. It has been hypothesized that the G allele is beneficial with regard to social traits as it has been associated with enhanced general sociality (Kogan et al., 2011) and sensitive parenting (Bakermans-Kranenburg and van Ijzendoorn, 2008).

The dominant hypotheses that both oxytocin and *OXTR* rs53576 G allele enhance sociality in humans have been challenged by more recent studies associating oxytocin and the G allele with non-prosocial outcomes and maladaptive behaviors. For instance, oxytocin administration heightens envy during a gamble game and can induce antisocial and aggressive behavior (Shamay-Tsoory et al., 2009), whereas plasma oxytocin levels have been positively associated with relational distress in women (Tabak et al., 2011). A significant effect of rs53576 on conduct problems was also found, with GG homozygotes showing higher levels of conduct problems compared to AA homozygotes (Smearman et al., 2015). However, a recent meta-analysis including more than 17,000 participants, reported no association of rs53576 with personality, social behavior, or psychopathology, and further that the absence of association was not due to clinical status, age, or sex (Bakermans-Kranenburg and van Ijzendoorn, 2014).

The contradictory results of the association of the rs53576 *OXTR* genotypes and oxytocin hormone with behaviors and symptoms may be due to the failure to take account of environmental factors. Consistent with the social salience hypothesis (Belsky et al., 2009; Tabak, 2013; Shamay-Tsoory and Abu-Akel, 2016), we have identified five studies reporting that negative and positive environmental factors modify outcomes only among G carriers or GG homozygotes. G allele carriers who had experienced maltreatment in childhood have been observed to show heightened emotional dysregulation (Bradley et al., 2011) and elevated levels of depression (McQuaid et al., 2013), while maltreated GG carriers showed higher levels of internalizing symptoms (Hostinar et al., 2014). Additionally, two studies have shown that positive environmental factors also modify the association of rs53576 *OXTR* with outcomes. Among children at varying levels of risk for autism, higher affective mutuality in early parent–child interactions was associated with higher empathy levels among GG homozygotes, but not A allele carriers (McDonald et al., 2016). Similarly, among G allele carriers, but not AA homozygotes, a more positive childhood family environment was associated with higher levels of resilient coping and positive affect in adulthood (Bradley et al., 2013). Another study showed however that rs53576 *OXTR* did not modify the association of childhood maltreatment with anxiety or depression (Tollenaar et al., 2017). In a recent meta-analysis, few studies included measures of environmental factors thereby preventing any calculations to determine whether the associations of *OXTR* genotypes with clinical phenotypes varied as a function of environmental factors (Bakermans-Kranenburg and van Ijzendoorn, 2014).

Previous studies suggest that oxytocin and *OXTR* in general, and *OXTR* rs53576 in particular, play distinct roles in females and males (Taylor et al., 2010; Tost et al., 2010; Gabor et al., 2012; Wang et al., 2014). Furthermore, ethnic differences in *OXTR* rs53576 genotype frequencies have been reported (Bakermans-Kranenburg and van Ijzendoorn, 2014). Thus, studies that take account of environmental factors, sex, and ethnicity are needed to further elucidate the role of *OXTR* rs53576 in human behavior.

Conduct disorder (CD) is the most common pediatric mental disorder affecting approximately 12% of boys and 7% of girls (Nock et al., 2006) prior to age 15. CD is characterized by a repetitive and persistent pattern of behavior in which the basic rights of others or major age appropriate social norms or rules are violated. The diagnosis includes symptoms of aggressive behavior (aggression to people and animals, destruction of property) and symptoms not indexing aggressive behavior (deceitfulness or theft, serious violations of rules). Not only the prevalence, but also the symptom presentation differs in boys and girls, with girls showing a later age of onset and less physically aggressive behavior than boys (Lahey et al., 2006; Brennan and Shaw, 2013). Large proportions of children with CD experience maltreatment (Afifi et al., 2009). A review of studies on childhood maltreatment has proposed that the occurrence of childhood maltreatment may be more important than its form, severity or duration (Briere and Jordan, 2009). CD is moderately heritable (Rhee and Waldman, 2002). One study has shown higher levels of conduct problems among *OXTR* rs53576 GG homozygotes compared to AA homozygotes (Smearman et al., 2015). To the best of our knowledge, no study has determined whether the association of maltreatment with conduct problems is modified by *OXTR* rs53576 genotypes and whether this differs in males and females.

The present study aimed to determine whether the association of maltreatment with conduct problems was modified by *OXTR* rs53576 in males and females. The study examined 1591 teenagers who voluntarily, and anonymously, responded to questionnaires and provided saliva samples for DNA extraction.

## Materials and Methods

### Participants

Teenagers born between 1997 and 1999, and their parents, from Västmanland, Sweden, were recruited in 2012 (Vadlin et al., 2015). All were initially contacted by mail and invited to participate in the study. Forty percent of those contacted agreed to participate and signed consents after the study was fully explained. The adolescents completed a questionnaire asking about socio-demographic characteristics, experiences in the family, and their mental health. Ninety-two percent of the adolescents also provided a saliva sample for DNA extraction. Those who had lived in Sweden for less than 5 years, those with protected identities, and those with mental disabilities were excluded. The final sample included 691 boys and 900 girls.

This study was carried out in accordance with the recommendations of the ethics committee of the medical faculty of Uppsala University with written informed consent from all subjects. All subjects gave written informed consent in accordance with the Declaration of Helsinki. The protocol was approved by the ethics committee of the medical faculty of Uppsala University, dnr 2012/187.

### Measures

#### Conduct Problems

Participants completed questionnaires to report on their own conduct problems. Response alternatives, never (0), once (1), 2–4 times (2), 5–10 times (3), more than 10 times (4), were summed to create a conduct problems score. The score was based on 26 answers, 18 answers indicating non-aggressive conduct problems and eight indicating aggressive behavior: how often, if ever have you 1…broken the rules at home? 2…broken the rules at school? 3…played with fire, so it caught fire for real? 4…taken goods in a store, shop or kiosk without paying? 5…deliberately smashed or wrecked windows, streetlights, benches, or other public things? 6…taken money at home that did not belong to you? 7…without permission painted graffiti, or scrawled on, for example, a public wall? 8… broken into a house, shop, store, kiosk, or other building with the intention to steal things? 9…stolen anything from another person’s pocket or bag? 10…sold or bought something you knew was stolen? 11…stolen a bike? 12…stolen a car? 13…stolen a moped, motorbike, or motor scooter? 14…dodged payment at the movies or a café, on a bus, train, or similar? 15…been involved in breaking into and stealing something from a car? 16…by yourself broken into and stolen something from a car? 17…driven a moped, motorbike, or car while being drunk? 18…been taken by the police for something you did? How often, if ever have you 1…played violent games with animals, where the animal been hurt? 2…threatened or forced someone to give you money, cigarettes, or anything else? 3…been involved in a fight during your leisure time (not at school)? 4…carried a weapon (knuckle-duster, baseball bat, knife, switch-blade, or similar) at school or during your leisure time? 5…hit/kicked someone so hard he/she needed medical attention? 6…deliberately hurt someone with a knife, switch-blade, knuckle-duster, or similar? 7…been involved in threatening another person to do something he/she did not want to, 8…by yourself threatened another person to do something he/she did not want to?

In males, the mean number (standard deviation) of the conduct problems score was 6.3 (6.6) and the median was 5. The corresponding statistics in females were 4.4 (5) and 3.

#### Ethnicity

Ethnicity was defined as Scandinavian – both parents born in Scandinavia, or not Scandinavian – at least one parent not born in Scandinavia.

#### Maltreatment

Maltreatment by parents was reported by the adolescents in response to four questions: “Have there ever been severe, heart-rending quarrels between your parents?”; “Has either of your parents ever pushed, beaten or used any other kind of violence against the other?”; “Has either of your parents ever pushed or beaten you, or used any other kind of violence against you?”; and “Have you ever been treated badly psychologically (for example, taunted or scorned) by either of your parents?” Answers ranged from “No/has not occurred” coded as 0 to “Yes/every or almost every day” coded as 6. This is a shortened version of a previously reported family conflict scale (Nilsson et al., 2006, 2014). Cronbach’s alpha was 0.68. The continuous maltreatment variable was used in initial and final statistical analyses. In order to illustrate interactions, the variable was dichotomized. Maltreatment was reported by 28.8% of the boys and 29.2% of the girls.

### DNA

DNA was extracted from 200 μl of saliva collected with the Oragene self-collection kit (DNA Genotek^®^) using the silica-based Kleargene DNA extraction method. Genotyping analyses of the SNP rs53576 were performed using the Kbioscience Allele-Specific Polymorphism assay based on competitive allele-specific PCR and bi-allelic scoring (LGC^®^). No-template control samples were included to enable the detection of contamination or non-specific amplification. Participants were classified as AA, AG, or GG. In all analyses, a G-dominant approach was applied in line with the majority of recent studies.

### Statistical Analysis

In gene-by-interaction studies, the statistical models applied are simplified outlines of theoretical models and biological phenomena and should thereby be considered as tools based on theory and previous knowledge (Hayes and Rockwood, 2017). Thus, in the present study, analyses were performed separately for males and females as (a) oxytocin has a stronger influence in female than male behavior (Taylor et al., 2010; Gabor et al., 2012), (b) estrogens affect the transcription of the *OXTR* gene (Choleris et al., 2008), and (c) sex-specific associations of *OXTR* rs53576 and brain limbic structures and connectivity have been reported (Tost et al., 2010; Wang et al., 2014).

It has also been recommended that a gene-by-environment interaction term should be tested using a full model, that is a model that includes the gene, the environmental factor, the gene-by-environment interaction and confounding factors (Keller, 2014). Our initial models investigated associations of the *OXTR* rs53576, maltreatment, and ethnicity with conduct problems score. In the final models, in order to control for the potential confounding effect of ethnicity we followed the recommendation of Keller (2014) and entered ethnicity, the ethnicity-by-gene, and the ethnicity-by-environment interaction terms in the same model along with the gene-by-environment interaction. Thus, our final models included *OXTR* rs53576, maltreatment, the *OXTR*-by-maltreatment interaction term, ethnicity, the ethnicity-by-*OXTR* interaction, and the ethnicity-by-maltreatment interaction. The level of significance for main and interaction terms was set at 0.05. In order to interpret the direction of significant gene-by-environment interaction terms, family maltreatment was dichotomized and follow up analyses were conducted.

In the present study, given that a general population sample was studied, the distribution of the conduct problems score was highly positively skewed. When the distribution of the dependent variable is highly skewed, the median is considered a more appropriate measure of central tendency than the mean which is sensitive to outliers. Thus, the analysis was not conducted with statistical methods comparing adjusted means, such as the Analysis of Covariance. It has been shown that when a highly skewed dependent variable is compared across groups, an ordinary least-squares regression can result in loss of power and thereby false-negative results (type II error) or even false-positive results (type I error) (McGreevy et al., 2009). A common approach used to treat non-normally distributed data is the transformation of the dependent variable. However, transformations may fail to normalize the data and results based on transformed data are difficult to interpret. In such cases, adjusted medians obtained from median regression provide more robust results (Beyerlein, 2014).

In the present study, due to the highly positively skewed distribution of the dependent variable, analyses were conducted using median regression in order to model the associations between the independent variables and the median of the dependent variable. Median regression is used to obtain adjusted medians and it is a special case of quantile regression (McGreevy et al., 2009; Beyerlein, 2014). Quantile regression has been mainly introduced during the past two decades and although it is currently implemented in many standard statistical packages, it is still surprisingly underused in medical research (Beyerlein, 2014). However, recently, the value of the method has been recognized and this method has been used in genetic studies (Briollais and Durrieu, 2014). The computation of the regression coefficients in median regression differs from linear regression as it is based on minimizing the sum of weighted absolute residuals instead of squared residuals (Beyerlein, 2014). Importantly, median regression allows adjustment for potential confounders and calculation of interaction terms and further the median regression coefficient quantifies the shift of the median of the dependent variable distribution by 1-unit increase in the independent variable (Beyerlein, 2014).

Generalized linear models (linear with robust estimator) were also applied for the models that included the interaction terms in order to confirm the median regression results. Median regressions were conducted with the statistical software Stata 14.1, whereas all the other analyses were conducted with the statistical software IBM SPSS Statistics 23.

## Results

Comparisons of AA homozygotes and G allele carriers are presented in **Tables 1, 2**. Among both males and females, AA homozygotes and G allele carriers did not significantly differ as to maltreatment, conduct problems score, and ethnicity. No significant deviation from Hardy-Weinberg equilibrium was detected (chi-square = 0.497 with *p* = 0.481 in males; chi-square = 2.240 with *p* = 0.135 in females).

**Table 1:** Comparisons of characteristics of AA homozygotes and G allele carriers among male participants.

Males (*N* = 691)	AA homozygotes	G allele carriers	Statistics
No. of participants	94	597	
% Participants	13.6	86.4	
% Scandinavians	72.3	80.4	X^2^(*N* = 691) = 3.216, *p* = 0.073
Mean (standard deviation) score maltreatment	0.628 (1.312)	0.694 (1.376)	*F*(1,689) = 0.188, *p* = 0.665
Mean (standard deviation) conduct problems score	6.628 (7.386)	6.235 (6.521)	*F*(1,689) = 0.284, *p* = 0.594
Median conduct problems score	4	5	0.978^1^

*^*1*^Independent samples Mann–Whitney *U*-test.*

**Table 2:** Comparisons of characteristics of AA homozygotes and G allele carriers among female participants.

Females (*N* = 900)	AA homozygotes	G allele carriers	Statistics
No. of participants	91	809	
% Participants	10.1	89.9	
% Scandinavians	74.7	80	X^2^(*N* = 900) = 1.381, *p* = 0.240
Mean (standard deviation) score maltreatment	0.846 (1.994)	0.842 (1.861)	*F*(1,898) = 0.000, *p* = 0.983
Mean (standard deviation) conduct problems score	3.868 (4.198)	4.449 (5.038)	*F*(1,898) = 1.121, *p* = 0.290
Median conduct problems score	3	3	0.385^1^

*^*1*^Independent samples Mann–Whitney *U*-test.*

Among males, the initial statistical model (**Table 3**) including *OXTR*, maltreatment, and ethnicity, detected no association of *OXTR* with the median conduct problems score. Maltreatment and Scandinavian ethnicity were associated with higher median conduct problems score. Specifically, the interpretation of the regression coefficients is that there is an increase of the adjusted median conduct problems score of one unit by 1-unit increase in maltreatment and further there is a decreased adjusted median conduct problems score of two units among non-Scandinavians compared to Scandinavians. **Table 4** presents the results of the final statistical model. The regression coefficients of the interaction terms in the final model are not intuitively interpretable and in case of significant *OXTR*-by-maltreatment interaction term, follow-up analysis within each genotype group should be applied, however among males, the interaction of *OXTR* and maltreatment was not associated with the median conduct problems score (*p* = 0.539). This final model among male participants was also tested with generalized linear model (linear with robust estimator) showing also no association between the *OXTR*-by-maltreatment term and conduct problems score (*p* = 0.917).

**Table 3:** Results of median regression of the associations of median conduct problems score with *OXTR* rs53576, maltreatment, and ethnicity among males and females.

	Median conduct problems score					
	Males (*N* = 691)			Females (*N* = 900)		
	Coefficient	*SE*	*p*-value	Coefficient	*SE*	*p*-value
*OXTR*	0	0.645	1.000	1	0.552	0.071
Maltreatment	1	0.162	**<0.001**	1	0.090	**<0.001**
Ethnicity	–2	0.547	**<0.001**	–1	0.415	**0.016**

*p-values < 0.05 are shown in bold.*

**Table 4:** Results of median regression of the associations of median conduct problems score with *OXTR* rs53576, maltreatment, *OXTR* rs53576 × maltreatment, ethnicity, ethnicity × *OXTR* rs53576, and ethnicity × maltreatment among males and females

	Median conduct problems score					
	Males (*N* = 691)			Females (*N* = 900)		
	Coefficient	*SE*	*p*-value	Coefficient	*SE*	*p*-value
*OXTR*	–1.22e-15	0.845	1.000	0	0.676	1.000
Maltreatment	2.333	1.060	0.028	–0.6	0.607	0.323
*OXTR* × maltreatment	–0.333	0.542	0.539	0.8	0.305	**0.009**
Ethnicity	1	3.010	0.740	–3	2.571	0.244
Ethnicity × *OXTR*	–1	1.597	0.531	1	1.344	0.457
Ethnicity × maltreatment	–0.667	0.378	0.078	–0.2	0.195	0.306

*p-values < 0.05 for the interaction terms are shown in bold.*

**FIGURE 1:**
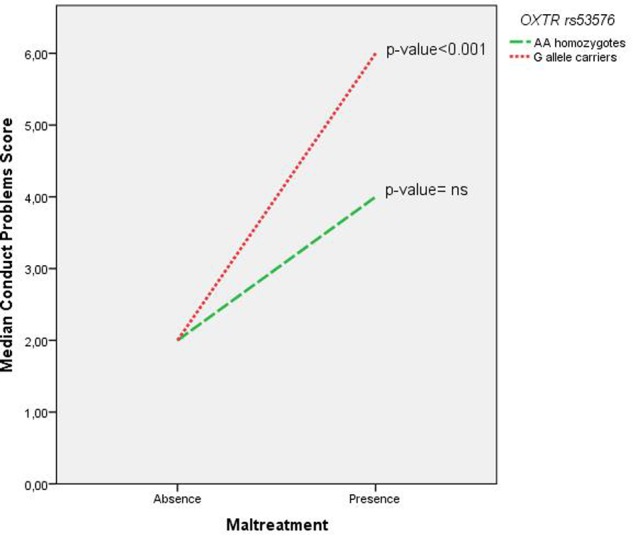
Interpretation of the significant interaction of *OXTR* rs53576 and maltreatment on median conduct problems score (*p* = 0.009) among females with follow-up analysis. In the absence of maltreatment there was no genotype effect. Among G allele carriers, there was a significant increase in median conduct problems score as a function of maltreatment (*p* < 0.001), whereas among AA homozygotes, maltreatment did not significantly affect the median conduct problems score (*p* = non-significant). The analyses were conducted with median regressions.

Among females, the initial model (**Table 3**) showed no significant association of *OXTR* with the median conduct problems score. Maltreatment and Scandinavian ethnicity were associated with higher median conduct problems score. Specifically, the interpretation of the coefficients is that there is an increase of the adjusted median conduct problems score of one unit by 1-unit increase in maltreatment and further there is a decreased adjusted median conduct problems score of one unit among non-Scandinavians compared to Scandinavians. The final model, presented in **Table 4**, showed that the interaction of *OXTR* and maltreatment was significantly associated with the median conduct problems score (*p* = 0.009). Follow-up analyses were conducted to assess the direction of the interaction revealing that in the absence of maltreatment there was no genotype effect. The median conduct problems score significantly increased as a function of maltreatment among G allele carriers (*p* < 0.001). By contrast, among AA carriers, no significant change of the conduct problems median score as a function of maltreatment was found (*p* = 1.000). These associations are illustrated in **Figure 1**. This final model among female participants was also tested with generalized linear model (linear with robust estimator) showing also a significant *p*-value for the interaction term (*p* = 0.009).

## Discussion

The present study examined a large general population sample and showed that among females carrying the G allele, those who experienced maltreatment presented a significantly higher conduct problems score than those who had not been maltreated, whereas among AA homozygous females, maltreatment did not modify the level of conduct problems. In males, the level of maltreatment was associated with conduct problems independently of genotype.

The detrimental effect of the G allele and the protective effect of the A allele among female participants, found in the present study, partially concur with the results from recent studies reporting a detrimental effect of the G allele coupled with negative environmental factors on clinical phenotypes. These studies analyzed males and females together not reporting sex-specific results. For example, one study in Caucasian youth reported that G allele carriers who experienced high levels of social stress showed higher levels of antisocial behavior, whereas in AA homozygotes the level of social stress was not associated with antisocial behavior (Smearman et al., 2015). Another study in youth of different ethnicities reported higher levels of depressive symptoms among G allele carriers, but not AA carriers, who had experienced high levels of childhood maltreatment (McQuaid et al., 2013). A study of African–American adults showed that childhood adversities, more specifically lack of warmth or stability in the family, negatively affected resilient coping style in G allele carriers, whereas AA individuals did not show such an association (Bradley et al., 2013). Another study in adolescents of different ethnicities reported that maltreatment in early childhood was associated with internalizing symptoms among GG homozygotes only (Hostinar et al., 2014). Another study reported that in children at varying risk for autism, lower quality of parent–child interaction early in life was associated with lower empathy levels in GG homozygotes, but not among A allele carriers (McDonald et al., 2016). And one other study of African–American adults showed that only G allele carriers showed heightened emotional dysregulation after childhood maltreatment (Bradley et al., 2011). To the best of our knowledge, there was only one study with inverse results, reporting that the A allele did not have a protective effect. Specifically, there was a stronger negative effect of a less secure attachment style on social anxiety among A allele carriers (Notzon et al., 2016).

These studies along with the results of the present study, with the exception of the study by Notzon et al. (2016), provide evidence that the association of negative environmental factors with behaviors and symptoms are genotype-dependent with the A allele conferring resilience. This interpretation would be consistent with the social salience hypothesis according to which specific genotypes or other factors such as neuropeptide levels are associated with a heightened salience, resulting in an increased sensitivity to the environment (Belsky et al., 2009; Tabak, 2013; Shamay-Tsoory and Abu-Akel, 2016).

Findings from the present study indicate the necessity of taking account of sex when investigating associations of the interactions of *OXTR* rs53576 genotypes and environmental factors with antisocial behavior. This may also be true when investigating the role of *OXTR* and environmental factors with other clinical phenotypes. Our sex-dependent results are in line with evidence from animal and human studies showing that oxytocin is more influential in female than male behavior, whereas arginine vasopressin, a neuropeptide with a similar molecular structure, may play a greater role in males than females (Taylor et al., 2010; Gabor et al., 2012). The interaction of *OXTR* rs53576 and oxytocin plasma levels is associated with emotional stress responses only among females (Moons et al., 2014). Further, sex-specific associations of *OXTR* rs53576 and brain limbic structures and connectivity have been reported. In one study, Han Chinese female AA carriers showed reduced amygdala volumes and reduced functional coupling between amygdala and prefrontal cortex bilaterally compared to G allele carriers, whereas in males there was no such associations (Wang et al., 2014). In another study with Caucasian participants, the A allele was associated with increased right gray matter amygdala volume in males and reduced right amygdala volume in females (Tost et al., 2010). As estrogens promote the rate of transcription of the *OXTR* gene (Choleris et al., 2008) and the release of oxytocin from hypothalamic neurons (Akaishi and Sakuma, 1985), the systematic differences in estrogen levels between males and females may explain, at least in part, the sex-specific associations of oxytocin and *OXTR* with different phenotypes.

The protective effect of the A allele or inversely, the detrimental effect of the G allele on human behavior in adverse environments, observed in the present study and reported in previous investigations, provides further support for the social salience hypothesis of oxytocin (Tabak, 2013; Shamay-Tsoory and Abu-Akel, 2016). The results of these studies may be interpreted as showing that the G allele provides heightened salience, or increased sensitivity, to the environment, whereas the A allele confers resilience. Heightened social salience among G allele carriers would increase the likelihood of adaptive functioning in positive, supportive environments, but would be a liability when negative environmental factors such as maltreatment occur.

Resilience refers to the capacity of an individual to withstand or recover from significant adversities (Masten, 2007). Previous studies have shown that among physically abused children, children’s internalizing well-being and prosocial behavior as well as caregivers’ well-being were associated with lower levels of aggressive behavior (Holmes et al., 2015). Further, both a positive family environment (Bradley et al., 2013) and individual levels of sensation seeking have been reported to promote resilience (Engel-Yeger et al., 2016). While sensation seeking has been associated with attachment (Jerome and Liss, 2005), its role in resilience to childhood maltreatment requires further study.

Oxytocin increases the salience to both positive and negative social cues, and genotypic variation in *OXTR*, associated with an increased sensitivity to endogenous oxytocin, may also increase the sensitivity to both negative and positive environmental factors (Tabak, 2013). However, the functionality of the *OXTR* rs53576 and its putative effect on oxytocin receptor sensitivity to endogenous oxytocin is presently unknown. Additionally, rs53576 is located in a non-coding region of the gene (intron 3) and it is not known whether the interaction identified in the present study is attributable to rs53576 or to other genetic loci in strong linkage disequilibrium with rs53576. One study showed an involvement of *OXTR* intron 3 in transcriptional suppression or downregulation of the gene (Mizumoto et al., 1997) suggesting that the observed interaction may be attributed to the investigated SNP.

The effect of *OXTR* genotypes on human behavior is likely mediated by intermediate neurobiological phenotypes in the central nervous system, such as structural and functional brain alterations. A recent study reported gray matter reduction in the bilateral ventral striatum with increasing childhood trauma questionnaire scores among GG carriers only (Dannlowski et al., 2016). We thereby hypothesize that the detrimental effect of the G allele on conduct problems in the presence of maltreatment may be mediated by a bilateral ventral striatum gray matter reduction. Another candidate structure mediating the genotype-dependent maltreatment effect on conduct problems is the amygdala. The amygdala plays an important role in human social cognition and oxytocin release and binding occur in the amygdala (Vargas-Martinez et al., 2014). Furthermore, reduced amygdala gray matter volume has been associated with both conduct disorder (Noordermeer et al., 2016) and childhood maltreatment (Hanson et al., 2015; van Velzen et al., 2016). No studies have searched for *OXTR* rs53576 genotype-dependent associations between maltreatment and amygdala gray matter volumes.

Childhood maltreatment is a complex phenomenon leading to multiple negative outcomes including an increased risk of conduct problems as shown in previous studies and confirmed in the present study. Depending on the type and the severity of maltreatment and other family, parent, and child characteristics, effective treatments aimed at reducing child conduct problems focus on parenting and/or the parent–child relationship (Toth et al., 2013). For instance, child–parent psychotherapy comprises a standardized treatment promoting attachment security and preventing behavioral problems (Cicchetti et al., 2006; Toth et al., 2013). In a multi-site study in Sweden, parent management training programs were shown to reduce child conduct problems (Stattin et al., 2015), and further, in a 10-year study, multi-systemic family intervention focusing on parent, child, home and school environments effectively reduced conduct problems and antisocial behavior in high risk children (Conduct Problems Prevention Research Group, 2007). Additionally, studies have shown that genotypes conferring sensitivity to environmental factors modify response to such interventions aimed at reducing conduct problems (Brody et al., 2009; Albert et al., 2015). To the best of our knowledge, no treatment trials of interventions aimed at reducing childhood conduct problems have measured the influence of *OXTR* rs53576 genotypes on outcomes. Based on the results of the present study, we hypothesize that among girls with conduct problems, those carrying the G allele would respond more positively than AA carriers to interventions aimed at reducing conduct problems.

The present study is characterized by a number of limitations. One, maltreatment and conduct problems were self-reported which may lead to differences in the accuracy of the recollections of maltreatment dependent on the level of conduct problems. Thus, the direct effect of maltreatment on conduct problems score found in the study may be to some extent a result of recall bias. By contrast, the validity of self-reports likely did not differ in participants carrying the different genotypes. We thereby conclude that the significant *OXTR*-by-maltreatment interaction that was associated with conduct problems in females is robust. Another possible limitation of the study was the high rate of refusal to participate. Selection bias occurs when there are systematic differences in characteristics between participants and individuals who refused to participate. Importantly, results of gene-by-environment interaction studies may not be influenced by selection bias as long as the genotype being studied does not influence the decision of whether or not to participate (Morimoto et al., 2003). We thereby consider that the risk that the gene-by-environment interactions observed are biased by self selection is low. Another limitation of the present study is the self-reported ethnicity. The study is characterized by several strengths including the relatively large sample, analyses conducted separately among females and males, and statistical analyses that were appropriate given the skewed distribution of the dependent variable. Further, this is one of the few gene-by-environment studies that have controlled for potential confounders following Keller’s (2014) recommendations. Additionally, another strength of the study may be that the participants provided self-reports and DNA anonymously. This may have encouraged them to provide accurate estimates of conduct problems and maltreatment.

## Conclusion

Among female *OXTR* rs53576 G allele carriers, those who experienced maltreatment presented significantly more conduct problems than those who had not been maltreated, whereas among AA homozygous females, maltreatment was not associated with the level of conduct problems. These results show again that similar adverse events such as childhood maltreatment are associated with different outcomes depending on genotype. However, in the case of *OXTR* rs53576 in males, the association of maltreatment with conduct problems was not modified by genotype.

## Author Contributions

DA and SH conceived and designed the study, planned the data analyses, interpreted the findings, and together wrote the first drafts of the manuscript. DA performed the statistical analysis. CÅ was responsible for the data collection and the preparation of all the data for analyses. EC participated in literature search and was responsible for DNA extraction. KWN contributed to the conception and design of the study, acquisition of data, analysis and interpretation of data. All authors critically revised several versions of the manuscript and approved the final version.

## Conflict of Interest Statement

The authors declare that the research was conducted in the absence of any commercial or financial relationships that could be construed as a potential conflict of interest.
